# Structural and molecular determinants of HIV-1 Gag binding to the plasma membrane

**DOI:** 10.3389/fmicb.2015.00232

**Published:** 2015-03-20

**Authors:** Jiri Vlach, Jamil S. Saad

**Affiliations:** Department of Microbiology, University of Alabama at Birmingham, Birmingham, AL, USA

**Keywords:** HIV-1, Gag, matrix, myristoyl, NMR, plasma membrane, PI(4,5)P_2_

## Abstract

Targeting of the Gag polyprotein to the plasma membrane (PM) for assembly is a critical event in the late phase of immunodeficiency virus type-1 (HIV-1) infection. Gag binding to the PM is mediated by interactions between the myristoylated matrix (MA) domain and PM lipids. Despite the extensive biochemical and *in vitro* studies of Gag and MA binding to membranes over the last two decades, the discovery of the role of phosphatidylinositol-4,5-bisphosphate [PI(4,5)P_2_] in Gag binding to the PM has sparked a string of studies aimed at elucidating the molecular mechanism of retroviral Gag–PM binding. Electrostatic interactions between a highly conserved basic region of MA and acidic phospholipids have long been thought to be the main driving force for Gag–membrane interactions. However, recent studies suggest that the mechanism is rather complex since other factors such as the hydrophobicity of the membrane interior represented by the acyl chains and cholesterol also play important roles. Here we summarize the current understanding of HIV-1 Gag–membrane interactions at the molecular and structural levels and briefly discuss the underlying forces governing interactions of other retroviral MA proteins with the PM.

## Introduction

**FIGURE 1:**
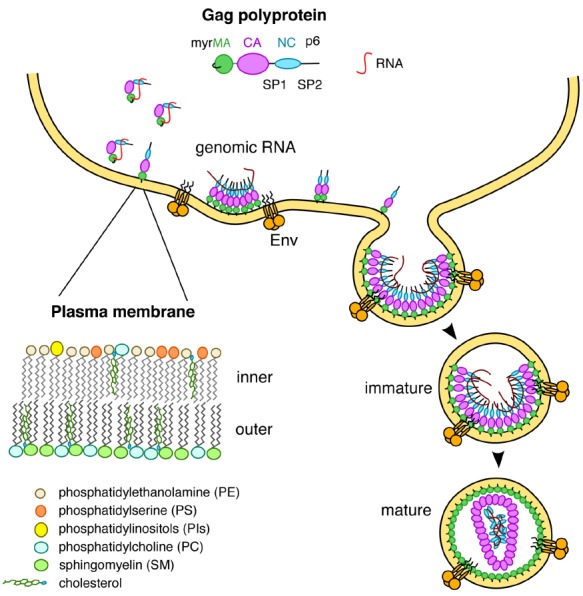
**The MA domain of Gag is involved in multiple interactions including RNA and lipids on the inner leaflet of the PM**.

Prior to assembly on the plasma membrane (PM), the human immunodeficiency virus type-1 (HIV-1) Gag polyprotein adopts a compact “folded over” conformation and exists in the monomeric or low-order oligomeric states (Datta et al., 2007a, 2011; Kutluay and Bieniasz, 2010; Kutluay et al., 2014). Whereas it is established that the nucleocapsid (NC) domain of Gag specifically recognizes motifs in the viral RNA genome for packaging (Jouvenet et al., 2009; Kutluay et al., 2014), there is compelling evidence that the matrix (MA) domain also binds to cellular RNA to prevent premature Gag targeting to intracellular membranes (Figure 1; Chukkapalli et al., 2010, 2013; Chukkapalli and Ono, 2011; Hogue et al., 2012; Inlora et al., 2014; Kutluay et al., 2014; Olety and Ono, 2014). Upon transport of Gag to the PM, the interaction of MA with RNA is exchanged for an interaction of MA with PM components (Figure 1; Chukkapalli et al., 2010, 2013; Chukkapalli and Ono, 2011; Hogue et al., 2012; Inlora et al., 2014; Olety and Ono, 2014). This molecular switch induces an extended conformation of Gag, leading to formation of high-order Gag oligomers on the PM (Datta et al., 2007a,b, 2011; Munro et al., 2014). The key to understanding this essential switch is elucidating at the molecular level the interaction of MA with specific PM components. Our current understanding of Gag–PM interaction is incomplete because of the lack of molecular details on how various membrane components contribute to the overall binding and how they control this molecular switch.

## Factors that Control HIV-1 Gag Assembly on the PM

For most retroviruses, assembly of the Gag proteins occurs on the PM of the infected cell (Ono et al., 2004; Jouvenet et al., 2006, 2008; Finzi et al., 2007; Welsch et al., 2007; Chukkapalli et al., 2008, 2010; Chu et al., 2010; Chukkapalli and Ono, 2011). The role of MA domain in Gag–PM binding is indispensable. Several factors can influence Gag–membrane binding including the myristoyl (myr) group, a conserved basic region in MA, protein multimerization, cellular RNA, and phosphatidylinositol-4,5-bisphosphate [PI(4,5)P_2_] (Ono and Freed, 1999; Ono et al., 2004; Jouvenet et al., 2006; Dalton et al., 2007; Chukkapalli et al., 2008, 2010, 2013; Alfadhli et al., 2011; Chukkapalli and Ono, 2011; Ghanam et al., 2012). The finding that Gag binds to membranes more efficiently than the isolated MA protein led to the hypothesis that the myr group is exposed in Gag and sequestered in the MA protein, which has become known as “the myr switch mechanism” (Zhou and Resh, 1996; Spearman et al., 1997; Hermida-Matsumoto and Resh, 1999; Ono and Freed, 1999; Paillart and Gottlinger, 1999). For over two decades, biochemical, *in vivo*, *in vitro*, and genetic data have provided invaluable insights on multiple factors that modulate Gag–membrane binding. However, only recently the molecular and structural determinants of this interaction have begun to emerge (Saad et al., 2006, 2007b, 2008; Shkriabai et al., 2006; Chukkapalli et al., 2008, 2010, 2013; Chukkapalli and Ono, 2011; Prchal et al., 2012; Vlach and Saad, 2013). Nuclear magnetic resonance (NMR) and analytical ultracentrifugation studies revealed that the myr group can adopt sequestered and exposed conformations in the MA protein, that the MA protein resides in monomer-trimer equilibrium, and that myr exposure is coupled with protein trimerization (Tang et al., 2004; Saad et al., 2007b). Exposure of the myr group is also modulated by other factors including the solution pH, inclusion of the CA domain, and binding of calmodulin (Tang et al., 2004; Fledderman et al., 2010; Ghanam et al., 2010). There is now convincing evidence that binding of RNA to MA prevents Gag from interacting with intracellular membranes (Chukkapalli et al., 2010, 2013; Chukkapalli and Ono, 2011; Inlora et al., 2014). As a consequence, RNA is considered as a negative regulator of Gag–membrane binding. Recent studies have shown that the MA domain binds almost exclusively to specific tRNAs in the cytosol (Kutluay et al., 2014). Incorporation of PI(4,5)P_2_ in membranes inhibits the interaction between MA and cellular RNA (Chukkapalli et al., 2010, 2013; Chukkapalli and Ono, 2011). Binding of Gag to membranes induces an extended conformation in the absence (Datta et al., 2011) or presence (Datta et al., 2007b) of inositol phosphates. Altogether, these studies indicate that particle assembly is regulated by coordinated interactions between the MA and NC domains of Gag with RNA and membrane lipids.

## Structural Studies of HIV-1 MA Binding to PM Lipids

**FIGURE 2:**
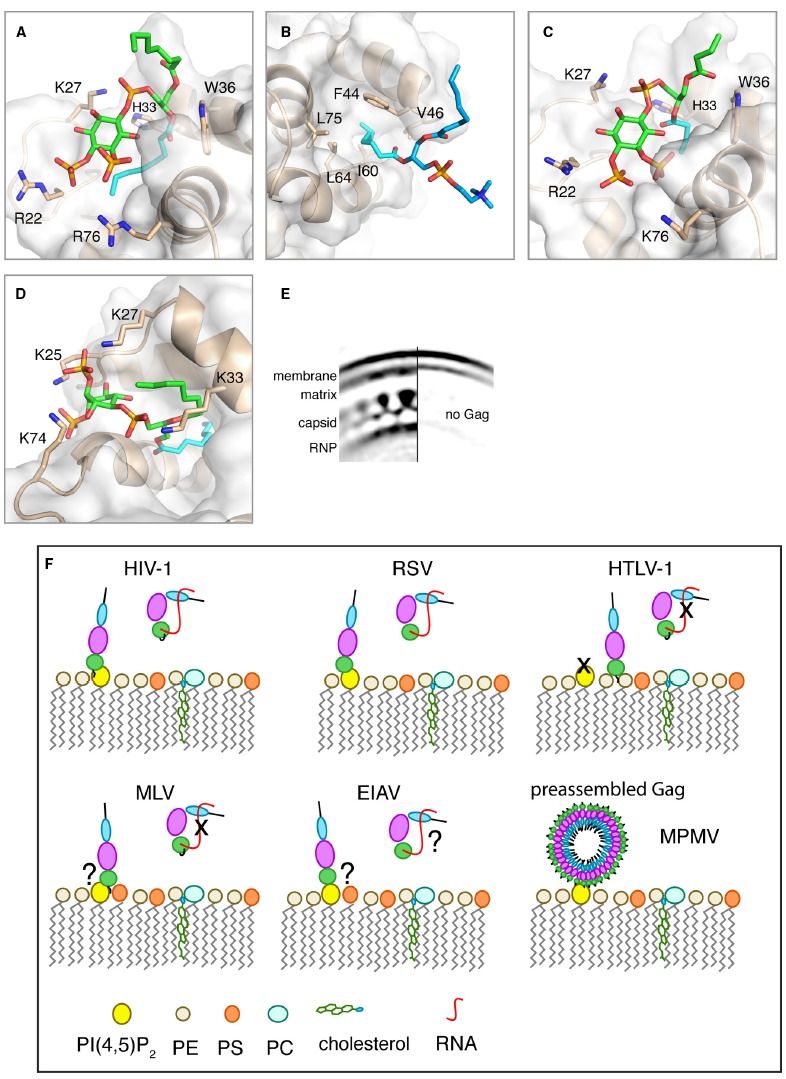
**(A–D)** Close-up views of structures of HIV-1, HIV-2, and M-PMV MA proteins bound to lipids. MA proteins are shown in ribbon and surface representations with residues implicated in binding shown as sticks. Phospholipids are shown as green [PI(4,5)P_2_] or blue (PC) sticks and their acyl chains involved in binding are shown in cyan. **(A)** HIV-1 myr(–)MA bound to di-C_8_-PI(4,5)P_2_ (PDB ID: 2H3V). **(B)** HIV-1 myr(–)MA bound to di-C_8_-PC (PDB ID: 2LYA). **(C)** HIV-2 MA bound di-C_4_-PI(4,5)P_2_ (PDB ID: 2K4I). **(D)** M-PMV MA bound to di-C_8_-PI(4,5)P_2_ structure provided by Prchal et al. (2012). **(E)** Cryoelectron microscopy reconstruction of HIV-1 immature particle in a section with (left) or without (right) Gag polyprotein present. Used with permission [Copyright (2009) National Academy of Sciences, USA (Briggs et al., 2009)]. **(F)** A schematic representation of potential mechanisms for Gag binding to the PM for different retroviruses. Gag binding to membranes is regulated by PI(4,5)P_2_ and RNA for some but not all retroviruses. Binding of Gag to membranes via the MA domain displaces RNA, which binds non-specifically to the basic region of MA. Data are not in agreement on the role of PI(4,5)P_2_ in MLV Gag binding to the PM. For EIAV, Gag binding to membranes appears to have no specific requirement for PI(4,5)P_2_ since other phosphoinositides may also play a role. For M-PMV, Gag assembly occurs in the cytoplasm prior to transport to the PM where MA specifically recognizes PI(4,5)P_2_.

Proper targeting of HIV-1 Gag to the PM is dependent on specific interactions between the MA domain and PI(4,5)P_2_ (Ono et al., 2004; Chukkapalli et al., 2008, 2010; Chukkapalli and Ono, 2011; Inlora et al., 2014). The most abundant form of PI(4,5)P_2_ contains saturated 18-carbon 1′ and 20-carbon unsaturated 2′ fatty acid chains (Dudley and Spector, 1986), which promote micelle formation in aqueous solution (Janmey et al., 1987). Interactions of HIV-1 Gag and MA with PI(4,5)P_2_ have been detected by mass spectrometric protein footprinting (Shkriabai et al., 2006). Titration of native PI(4,5)P_2_ into MA samples led to severe broadening and loss of NMR signals. Therefore, soluble analogs of PI(4,5)P_2_ with truncated 1′- and 2′-acyl chains (C_4_ or C_8_) have been used (Saad et al., 2006, 2007a, 2008). NMR studies have shown that soluble analogs of PI(4,5)P_2_ bind directly to HIV-1 MA, inducing a conformational change that promotes myr exposure (Saad et al., 2006). The solution structure of the MA–PI(4,5)P_2_ complex revealed that the 2′-acyl chain is inserted in a hydrophobic cleft, whereas the inositol group is packed against a highly basic region of MA (Figure 2A). The 1′-acyl chain, however, is not involved in binding and is exposed to solvent. It was suggested that PI(4,5)P_2_ can function as both an allosteric trigger for myr exposure and as a direct membrane anchor (Saad et al., 2006). The involvement of the acyl chain of PI(4,5)P_2_ in MA and Gag binding has been confirmed by surface plasmon resonance methods (Anraku et al., 2010). Based on the NMR studies, a structural model for Gag bound to PM has been proposed. In this model, MA is anchored to the membrane by the myr group and 1′-acyl chain of PI(4,5)P_2_, which bracket a patch of conserved basic residues that can interact with the negatively charged surface of PM (Saad et al., 2006). A molecular model of MA bound to native PI(4,5)P_2_ shows that a longer 2′-acyl chain (18 carbons) can be accommodated into the hydrophobic cleft (Saad et al., 2006). Structural studies have yet to determine the precise mode of MA binding to native PI(4,5)P_2_ within the context of a membrane bilayer.

In addition to PI(4,5)P_2_, other lipids have been implicated in HIV-1 Gag–membrane binding (Dalton et al., 2007; Ono, 2010a; Waheed and Freed, 2010; Chan et al., 2011; Chukkapalli and Ono, 2011; Chukkapalli et al., 2013). The affinity of Gag and MA to membranes is increased upon increasing the stoichiometry of phosphatidylserine (PS; Ehrlich et al., 1996; Zhou and Resh, 1996; Scarlata et al., 1998; Dalton et al., 2007; Chukkapalli et al., 2008, 2010, 2013; Alfadhli et al., 2009; Chan et al., 2011). NMR studies on HIV-1 MA binding to soluble analogs of PM lipids revealed that PS, phosphatidylethanolamine (PE) and phosphatidylcholine (PC) bind directly to MA via a distinct site that is adjacent to the PI(4,5)P_2_ binding site (Vlach and Saad, 2013). All three phospholipids interact with MA via sequestration of the 2′-acyl chain into a cavity formed by hydrophobic residues in helices α2 and α3, leaving the 1′-acyl exposed (Figure 2B). Surprisingly, the polar heads of PE, PS, or PC do not appear to play a major role in stabilizing the complex. Structural studies have been conducted with single lipids. It is likely that in a membrane environment the lipid polar heads will orient to make favorable electrostatic contacts with the basic domain of MA. Consistent with the hydrophobic nature of binding, the affinities of all three phospholipids to MA increased by two orders of magnitude upon extending the acyl chains from hexanoyl to octanoyl (Vlach and Saad, 2013). Intriguingly, the myr group is readily exposed when MA is bound to membrane mimetics such as micelles and bicelles in the absence of PI(4,5)P_2_. Exposure of the myr group does not appear to be triggered by an allosteric mechanism as in the case of PI(4,5)P_2_ binding, but rather by the mere presence of a lipid agglomerate. Based on these studies, we have proposed a trio engagement model by which HIV-1 Gag is anchored to the PM via the 1′-acyl chains of PI(4,5)P_2_ and PS/PE/PC lipids, and the myr group, which collectively bracket a basic patch projecting toward the polar leaflet of the membrane (Vlach and Saad, 2013). Exposure of the myr group by a membrane-like environment, independent of PI(4,5)P_2_ binding, was also observed in a reverse micellar system (Valentine et al., 2010). Spontaneous exposure of the myr group prior to association to membranes has also been observed by using coarse-grained simulation approaches (Charlier et al., 2014). The authors reported that insertion of the myr group into the bilayer is necessary for the orientation of MA to allow for the experimentally identified region of MA to interact with the PI(4,5)P_2_ head group. Flipping of the lipid acyl chain out of the membrane environment to the hydrophobic groove in MA was, however, not observed. Further studies are needed to answer yet unresolved questions: What is the actual trigger and mechanism of myr exposure *in vivo*? Are acyl chains of either PI(4,5)P_2_ or other phospholipids involved in MA binding to the PM? The Vogt lab has recently shown that Gag and MA strongly preferred lipids with both acyl chains unsaturated over those with one chain unsaturated (Dick et al., 2012). Furthermore, Gag can sense cholesterol in the membrane bilayer allowing for a more efficient Gag binding to membranes (Dick et al., 2012). However, it is not yet known whether cholesterol can bind directly to Gag. Recent studies have also suggested that interactions can occur between the isolated NC and p6 domains of Gag with membrane models (Solbak et al., 2013; Kempf et al., 2015). However, the biological significance of these interactions has yet to be established. Further studies are needed to determine the synergy and interplay between PM components and their role in modulating the conformational switch of HIV-1 Gag during assembly (Ono, 2010b).

## Other Retroviral Gag and MA Interactions with Lipids and Membranes

Like HIV-1, the site of HIV-2 assembly *in vivo* is also dependent on PI(4,5)P_2_ (Saad et al., 2008). Structural studies show that the HIV-1 and HIV-2 MA structures are very similar and that PI(4,5)P_2_ binds to both proteins in an identical manner (Figures 2A,C; Saad et al., 2008). However, in contrast to HIV-1 MA the position of the myr group in HIV-2 MA is less sensitive to factors that modulate myr exposure in HIV-1 MA such as protein concentration and binding of di-C_4_-PI(4,5)P_2_ (Saad et al., 2008). Data are not in agreement on the role of PI(4,5)P_2_ in other retroviruses. Previous studies have shown that murine leukemia virus (MLV) Gag targeting to the PM is mediated by PI(4,5)P_2_ and PS, suggesting a synergy between these two lipids to modulate Gag–PM binding (Hamard-Peron et al., 2010). The Ono laboratory, however, has recently shown that MLV Gag–PM binding is not dependent on PI(4,5)P_2_ (Inlora et al., 2014). The Gag proteins of Rous sarcoma virus (RSV) and equine infectious anemia virus (EIAV) lack the myr group and it is thought that Gag–membrane interaction is driven mainly by electrostatic interactions (Erdie and Wills, 1990; Provitera et al., 2000; Dalton et al., 2005; Chen et al., 2008; Fernandes et al., 2011). Association of RSV MA and Gag with liposomes of defined composition is dependent on the presence of a biologically relevant concentration of negatively charged lipids like PS (Dalton et al., 2005). The Vogt laboratory has shown that RSV Gag has no specific requirement for PI(4,5)P_2_ for PM association *in vivo* or liposome interaction *in vitro* (Chan et al., 2011). Parent and colleagues, however, have shown that depletion of intracellular PI(4,5)P_2_ and phosphatidylinositol-(3,4,5)-trisphosphate [PI(3,4,5)P_3_] levels impaired RSV Gag PM localization (Nadaraia-Hoke et al., 2013). It was suggested that differences are partially attributed to the sensitivity of assays utilized in these studies (Dick and Vogt, 2014). Most recent *in vitro* and *in vivo* studies from the Ono laboratory have shown that Gag–membrane binding via RSV MA is both PI(4,5)P_2_ dependent and is susceptible to RNA-mediated inhibition (Inlora et al., 2014). No structural studies are currently available on how RSV MA interacts with PM lipids.

The Carter laboratory has reported that a soluble analog of PI(4,5)P_2_ interacts directly with EIAV MA (Chen et al., 2008). More recently, it was shown that EIAV Gag is not only present on the PM but also in compartments enriched in phosphatidylinositol 3,5-biphosphate [PI(3,5)P_2_] (Fernandes et al., 2011). In contrast to HIV-1, release of EIAV particles is not significantly diminished by co-expression with 5-phosphatase IV, which may suggest that PI(4,5)P_2_ does not play a critical role in EIAV Gag assembly on the PM. Additional NMR studies have shown that phosphatidylinositol 3-phosphate [PI(3)P] binds to EIAV MA tighter than that of PI(4,5)P_2_ (Fernandes et al., 2011). Altogether, it is likely that EIAV Gag membrane targeting proceeds via a mechanism that is different from that of HIV-1, HIV-2, MLV, or RSV.

The Gag precursor of deltaretrovirus human T-lymphotropic virus type 1 (HTLV-1), Pr53Gag, localizes at the cell surface and intracellular compartments in HeLa cells (Le Blanc et al., 1999, 2002; Heidecker et al., 2004). It has been shown that subcellular localization of HTLV-1 Gag and VLP release are minimally or not sensitive to depletion of PI(4,5)P_2_ from the PM, suggesting that the interaction of HTLV-1 MA with PI(4,5)P_2_ is not essential for HTLV-1 Gag–membrane binding and particle assembly (Inlora et al., 2011, 2014). Single amino acid substitutions that confer a large basic patch rendered HTLV-1 MA susceptible to the RNA-mediated block, a phenotype similar to that observed for HIV-1 and RSV. It remains unknown whether other PM lipids play important roles in HTLV-1 Gag assembly as further studies are needed to identify the precise molecular requirements for HTLV-1 Gag binding to membranes.

Assembly and particle production of Mason-Pfizer monkey virus (M-PMV) is perhaps the most distinct from all cases discussed above. M-PMV is thought to catalyze the membrane envelopment of a preassembled spherical capsid shell to release infectious virions (Sfakianos and Hunter, 2003). Assembly of M-PMV capsids occurs in a pericentriolar region of the cytoplasm prior to transport to the PM for budding. It is thought that both MA and CA domains of M-PMV Gag interact with the PM (Sfakianos and Hunter, 2003). Initial evidence for a potential role of PI(4,5)P_2_ in M-PMV Gag–PM binding has been reported by Hunter and colleagues (Stansell et al., 2007). Depletion of PI(4,5)P_2_ from the PM led to dramatic decrease of particle release from M-PMV infected cells. Direct interaction between M-PMV MA and a soluble analog of PI(4,5)P_2_ has been detected (Prchal et al., 2012). The solution structure of M-PMV MA protein and its binding mode to di-C_8_-PI(4,5)P_2_ have been determined by NMR and molecular docking methods (for more details, see Prchal et al., 2012, 2014). The hallmark of the binding mode is the deep penetration of one of the C_8_ chains into a hydrophobic pocket formed between helices α1, α2, and α4 (Figure 2D). The position of the myr group of M-PMV MA was not affected by di-C_8_-PI(4,5)P_2_ binding and remained sequestered in the protein core. Interestingly, no binding was detected between the unmyristoylated MA protein of M-PMV and soluble analogs of PI(4,5)P_2_ (Prchal et al., 2012). The authors attributed this observation to the structural differences between MA and unmyristoylated MA, in which the hydrophobic cavity is absent in the latter. Altogether, the mechanisms of retroviral Gag assembly appear to be complex and require more detailed investigation at the molecular level (Figure 2F).

## Gaps in Our Understanding of Gag Binding to the PM and Future Directions

Structural studies of HIV-1 MA interactions with PI(4,5)P_2_, PS, PE, and PC have provided novel insights into the molecular mechanism of Gag assembly on the PM. These studies have utilized lipids with truncated acyl chains. The precise mode of binding of native lipids with longer chains to MA has yet to be elucidated. The lack of an atomic snapshot for MA when bound to membranes containing physiologically relevant lipid composition remains a major gap in our understanding of virus assembly. Investigation of the molecular rearrangements of Gag in the immature and mature HIV particles have heavily relied on cryoelectron microscopy (cEM) data. Although details of the hexameric CA lattice exist, cEM studies have not provided a clear picture of the membrane-associated MA domain of Gag in either of the immature or mature particles (Fuller et al., 1997; Briggs et al., 2006, 2009; Wright et al., 2007). cEM data show that the membrane bilayer is thicker in regions where the Gag lattice is present and that MA density appears to “penetrate” the membrane bilayer (Figure 2E; Briggs et al., 2009). Earlier cEM tomography studies have suggested that the membrane-bound MA domain lacks periodicity (Wright et al., 2007). We believe that future studies may benefit from hybrid structural and biophysical methods to construct a model of Gag and/or MA bound to membranes. These may include small-angle x-ray scattering, atomic force microscopy and high-resolution cEM. Understanding the molecular basis of Gag assembly will not only shed light on the assembly of HIV-1 particles but will likely provide insight into the control of assembly in other retroviruses that assemble at the PM. Such studies are of great importance as these interactions may serve as pharmacological targets to inhibit HIV assembly. Structure-based studies have identified small molecule inhibitors that bind to the PI(4,5)P_2_ site of HIV-1 MA and possess modest potency in reducing virus production (Zentner et al., 2013a,b; LaPlante et al., 2014). Although considered weak binders, these inhibitors may serve as a template for future studies aimed at the development of novel inhibitors of retroviral assembly.

### Conflict of Interest Statement

The authors declare that the research was conducted in the absence of any commercial or financial relationships that could be construed as a potential conflict of interest.

## Abbreviations

MA: matrix protein
NMR: nuclear magnetic resonance
PS: phosphatidylserine
PI(4,5)P_2_: phosphatidylinositol-4,5-bisphosphate.
